# Molecular war and peace: the concurrent path to antibiosis and antibiotic resistance

**DOI:** 10.3389/fmicb.2026.1851874

**Published:** 2026-06-10

**Authors:** Fernando Baquero, Rafael Cantón, Cristina Herencias, João A. Gama, Teresa M. Coque

**Affiliations:** 1 Servicio de Microbiología, Instituto Ramón y Cajal de Investigaciones Sanitarias (IRYCIS), Madrid, Spain; 2 Centro de Investigación Médica en Red – Epidemiología y Salud Pública (CIBERESP), Madrid, Spain; 3 Centro de Investigación Médica en Red – Enfermedades Infecciosas (CIBERINFEC), Madrid, Spain

**Keywords:** molecular wars, origin of antibiotic resistance, origin of antibiotics, origin resistance genes, prebiotic molecular evolution

## Abstract

The possibility of the emergence of proto-antibiotic and proto-resistance molecules in the prebiotic world, as primary elements involved in “molecular wars,” is examined in this conceptual review. Throughout the Earth’s early history, prebiotic chemical processes produced molecules that associated both randomly and persistently. Over time, those configurations that achieved greater stability were favored, their longevity effectively serving as a mechanism for prebiotic selection. Available chemical molecules or physical surfaces could stabilize and prolong the duration of certain aggregates, creating competition among them. Hypothetically, some aggregates could yield conformations capable of disrupting the assembly or stability of rival structures, thereby acting as proto-antimolecules and later evolving into proto-antibiotics in a primitive cellular scenario. Concurrently, some other molecular aggregates may deactivate such proto-antimolecules and antibiotics, acting as primitive mechanisms of resistance. Probably, both production and protection mechanisms tended to coalesce in multimolecular assemblies, ensuring the non-self-destruction of producers. Over a prolonged period, the chemical Thioester World, RNA World, and the biological Proto-Cellular World coexisted, and proto-organelles began to be influenced and protected by proto-antibiotics and proto-resistances. Antibiotic production and resistance remained associated, even at the stage of antibiotic polyketides, which progressively emerged in a more oxygenated landscape, with early biosynthetic pathways giving rise to contemporary ones, mainly in Actinomycota. This simultaneous action-and-reaction scenario provided an ecological equilibrium in which antibiotic molecules were not necessarily killer agents but rather regulatory signals within the microbiosphere, ensuring healthy bacterial interactions. The massive anthropogenic antibiotic production altered such an equilibrium, favoring an unbalanced resistance reaction through the massive diffusion of antibiotic resistance genes, now decoupled from antibiotic production and spreading across the microbial world, mostly carried in mobile genetic elements.

Life did not stop, and one had to live (Leo Tolstoy, War and Peace, 1863).

## Introduction: antibiotics and antibiotic resistance molecules as secondary metabolites

In cellular life, both antibiotics and antibiotic-resistance biomolecules originate from secondary metabolism. According to the classic view, bacterial secondary metabolites generally comprise small compounds, often including unusual chemical structures, which, unlike those arising from primary metabolism, are not strictly essential for the immediate growth or survival of an organism (Demain, 2007). However, secondary metabolites may be critically involved in microorganisms’ interactions with their natural environment, including bacterial interactions. The vast repertoire of secondary metabolites varies significantly across bacterial species, likely reflecting the evolutionary pressures and diversity of their environments. Notably, the basic chemical structures of most clinically relevant natural antibiotics, including *β*-lactams, glyco- and lipopeptides, macrolides, tetracyclines, or polymyxins, are non-ribosomally produced, but are synthesized by chromosomally encoded enzymatic complexes, the non-ribosomal peptide synthetases (NRPSs) (Miller and Gulick, 2016). These synthetases are located in secondary metabolite biosynthetic gene clusters (BGCs), as first described in *Streptomyces*. Within a single module, multiple catalytic domains are responsible for the incorporation of single molecules. The NRPSs enzymes are coordinated by communication (COM) domains, facilitating the appropriate assembly of these biosynthetic molecules. In addition, the NRPSs enzymes require a prior post-translational modification by a 4′-phosphopantetheinyl transferase, which acts on the thiolation domain of each module, to enable sequential amino acid binding.

These multi-modular ribosomally-produced enzymes catalyze, sometimes in combination with non-modular enzymes, the synthesis of antibiotics, frequently using a variety of standard and non-proteinogenic amino acid substrates (Wang et al., 2014). Interestingly, along the antibiotic biosynthetic pathway, BGCs may contain antibiotic-detoxifying (resistance) enzymes involved in the self-protection of the producer strain. However, another highly effective mechanism, involving transcriptional regulatory cascade regulators of BGCs expression, is frequently present in Actinomycetota (Bibb, 2005). Such regulators activate or repress BGCs’ biosynthetic activity, depending on environmental or cell-cycle conditions in the antibiotic-producing strain. That might explain why many antibiotic-producing strains lack resistance mechanisms; in fact, antibiotic production via secondary metabolism often occurs when primary metabolism is poorly active, thereby reducing self-damage. A particular type of these regulators comprises the *Streptomyces* Regulatory Antibiotic Proteins (SARPs) (Yan and Xia, 2024). Production of antibiotics can be modulated by quorum-sensing signals, such as butyrolactones, or by antibiotics themselves. Hypothetically, if the antibiotics cause structural or metabolic damage in the cell, the molecules released from this damage should be able to negatively regulate antibiotic biosynthesis. This may recall that cell wall bioactive molecules resulting from *β*-lactam damage can induce *β*-lactamases (Baquero et al., 2026). A stimulatory effect of BCG’s antibiotic biosynthetic activity might be beneficial if the population is involved in a competition with rival microorganisms. Finally, within a single protein molecule, combining antibiotic and antibiotic-resistance domains, resistance could serve as an intramolecular regulator, altering the molecular conformation and, consequently, the activity in response to environmental cues. Similar intramolecular regulatory effects have been detected in alarmones (Turnbull et al., 2019). The question we are examining in this work is the prebiotic origin of the molecular assemblies that gave rise to the ancestors of secondary metabolites involved in antibiotic production and antibiotic resistance. Prebiotic molecules involved in these action-reaction molecular interactions imply “molecular wars,” favoring the more stable molecular associations. The primordial-chemistry molecular weapons and defenses were evolving over billions of years to become biosynthetically produced and functionally improved. However, this evolutionary process occurred non-linearly, with periods of stagnation or acceleration during certain geological and biogeological periods.

## The common remote origin and evolution of antibiotics and antibiotic resistance molecules

Comparative genomics and environmental studies suggest that antibiotic biosynthetic genes and resistance determinants have been associated with one another for very long evolutionary periods within the same gene cluster (Allen et al., 2010). *Β*-lactamases existed 2,200 million years ago (Waglechner et al., 2021), thus deriving from molecules that precede the Great Oxygenation Event. Similarly, the molecularly and structurally related precursors of antibiotics are believed to be even older, a postulation supported by the presence of non-proteinogenic amino acids in peptide antibiotics found in meteorites and other primordial sources (Johnson et al., 2008). These hypotheses suggest that antibiotics and resistance are ancient features of microbial life, predating the anthropogenic actions (antibiotics) and reactions (resistance) by billions of years (Sengupta et al., 2013).

Expanding upon this framework, we hypothesize that primitive antibiotic-like and resistance-like molecules could have evolved in ancient prebiotic times through a sprawling, vast network of stochastic, pre-metabolic pathways with ephemeral, futile outcomes. These ancestral processes likely generated an enormous diversity of small bioactive compounds, probably exceeding the current bacterial population of the biosphere by an order of magnitude (Davies and Ryan, 2012). We can consider that a minute fraction of this “parvome” may have incidentally produced molecular combinations. Some of them are independently repeated due to chemical affinities in particular environments, a process that recalls re-plication, independent, reiterated formation of a molecular combination (Baquero et al., 2024). Applying the principles of natural selection, those of these aggregates with extended persistence over time, probably thermodynamically stable, should have been passively selected over dissipative assemblies. If some aggregates could have prevented the formation, robustness, and therefore persistence of others, they exerted an antibiotic-like destructuring effect, resulting in a kind of active selection. We can hypothesize that complexification with other molecules or aggregates could protect the destructuring aggregate from self-destruction while maintaining this effect against alien entities. Such a combination of attack and self-defense could, in theory, contribute to the persistence or robustness of particular molecular assemblies, recalling a selection-like process. Perhaps primitive antibiotics and pre-antibiotic resistance molecules evolved in ancient prebiotic times through a mesh of chemical diversification and coalescence of multiple stochastic pre-metabolic synthetic pathways, most of which were ephemeral and futile, that is, without functional or evolutionary consequences. As noted earlier, a very small number of these molecules may have served as precursors to antibiotics and related ancestral antibiotic-resistance molecules that were eventually co-selected. In both cases, the primitive may have evolved to a non-equilibrium self-assembly requiring capture of energy (Faran and Bisker, 2025). The selection of these precursors was probably based on the replication of a specifically ordered molecular sequence (Ruiz-Mirazo et al., 2014). Once more, among these, further selective events acted on the more robust, primarily based on their greater stability and thus their longer persistence over time.

Inorganic synthesis was ultimately replaced by biochemical reactions (Wang and Du, 2025). The origin of these ancient fixed and replicative sequences has been attributed to the prebiotic availability of auto-replicative RNA molecules, the ribozymes, which serve as backbones for substrate attachment (Chen et al., 2007). That was the birth of the biological information. Primitive metabolites could have emerged in such a way without preexisting protein machinery (Coggins and Powner, 2017). Proto-peptides contributed to the stabilization of RNA molecules, and vice versa (Frenkel-Pinter et al., 2020a,b). The strict requirement of ribonucleotidyl coenzymes in contemporary metabolic pathways suggests early intertwining of RNA with biochemical catalytic functions (Negrón-Mendoza et al., 2025). A quintessential example is found in protein synthesis: the critical enzyme peptidyl transferase is catalyzed not by another protein, but by the major 23S rRNA component of the large ribosomal subunit, a true ribozyme, recalling the ancient and modern association between ordering and catalyzing ribozymes and elements from metabolic pathways (Lilley, 2015; Lazcano and Miller, 1996). Such ancestral yet conserved pathways include those that may have given rise to precursors and to molecules involved later in antibiotics and antibiotic resistance. Many of their key structural monomer components were available in the ancient chemosphere. That is supported by the detection of non-proteinogenic amino acids and purine nucleobases in asteroids and meteorites (Parker et al., 2022; Johnson et al., 2008). Julian Davies proposed years ago that antibiotics may be among the oldest biomolecules (Amábile-Cuevas, 2003) and could have played a role as effectors of RNA catalytic reactions (Davies et al., 2007). In parallel, primordial antibiotic-resistance biomolecules likely date back billions of years and could have evolved from prebiotic molecules, altering the effects of these primitive antibiotics, which act not on cells but on molecular assemblies. However, such an evolutionary process was discontinuous in time, depending on the changing environmental conditions on the Planet.

## Non-linearity periods in evolution of antibiotic production and resistance

Evolutionary rates of antibiotic production and resistance were discontinuous over time and likely spatially heterogeneous. This nonlinear progression can be illustrated by the shifting influence of inorganic molecules throughout Earth’s history, including metal ions and oxygen. A critical environmental factor was the dramatic shift in oxygen availability. In prebiotic times, oxygen levels were less than 0.001% of present atmospheric levels (21%) (Carver, 1981). Due to oxygen’s high redox potential, its presence, as molecular oxygen and reactive oxygen species, can facilitate electron removal, breaking chemical bonds in primordial molecules. Consequently, its absence was probably a precondition for the synthesis and aggregation of molecules that involved electron removal and bond breaking in ancestors of proto-antibiotics via prebiotic chemistry, likely operative ca. 4 billion years ago. Although oxygen was present at low concentrations before the “great oxygenation event” (ca. 2.4 billion years ago), this event marked a major shift in Earth’s history (Olejarz et al., 2021), opening a quite different molecular evolutionary landscape based on biochemical networks (Raymond and Segrè, 2006). As mentioned above, we cannot rule out that oxygen availability, electron capture, and their influence on the emergence of pre-biochemical processes began earlier, on a much smaller scale (He et al., 2023). In fact, biochemical cycles might have been locally fostered by Earth’s mechanical biogeochemistry, essentially driven by piezoelectric processes convert mechanical forces into biochemical energy (Meng et al., 2025). In contrast to the anoxic phase, oxygen reactivity was now instrumental in the condensation of the more stable among prebiotic molecular associations. Evolutionary forces started to act, facilitating stability, diversification, and finally replication of cooperative molecular interactions, resulting in proto-enzymes and proto-metabolism. The acceleration of interactive prebiotic chemistry was suddenly paving the way for proto-biochemical processes, such as the formation of polycyclic hydrocarbons. Oxygen initiated the process that underlies the bedrock of all metabolisms: inorganic carbon fixation into organic matter (Shih, 2015). This process occurred in a progressive way, predating the emergence of atmospheric oxygen simultaneously, novel molecular oligomers became accessible by oxygen degradation of prebiotic aggregates, such as prebiotic oligosaccharides. Oxygen availability was (is?) a powerful factor in generating diversity in polyketide metabolites. Oxidation could further introduce functional moieties and even rearrange the existing chemical structure. Guided by monooxygenase enzymes, oxygen can be involved in structure editing of metabolites, either by carbon-to-oxygen swaps, carbonyl deletions with ring contraction and fully carbocyclic edits, carbon-to-nitrogen swaps, and skeletal reorganizations that retain the atomic formula but alter the connectivity (Ikonnikova et al., 2026). Flavoprotein monooxygenases were possibly proto-enzymes already present in the first cells, in the last universal common ancestor of all cells (LUCA) (Mascotti et al., 2015) and deeply involved in polyketide biosynthesis (Zhang and Ge, 2024). Monooxygenases are involved in methylations, glycosylations, and reductions, allowing the biosynthesis of polyaromatic antibiotic structures (Grocholski et al., 2012). Of course, the major contributor to the “Great Oxidation Event” that accelerated the building-up of polyketides, and thus antibiotics and antibiotic resistance molecules, was the widespread of oxygenic photosynthesis, using water to produce oxygen, dated 2.4–2.1 billion years ago (Sessions et al., 2009). However, the process of oxygenation was not a brief, rapid, punctuated event. Cyanobacteria, the more successful oxygenic bacteria, had slowly evolved under the influence of abiotic oxidants (Wu et al., 2023; Wang et al., 2016). Phylogenetic analysis traced the first organisms to use oxygen to 3.1 billion years ago, probably initially confined to small spatial sources, where oxygenases and oxidoreductases were already present (Jabłońska and Tawfik, 2021). These sources may have been located at mechanical piezoelectrical forces, hydrothermal vents, fissure-like hot springs on the seafloor, where suitable conditions of geothermal light, could have early bacteria allowed to use light for electron donor extraction, fostering the origin of the first, primitive enzymes, with structural pockets recognizing substrates, but much less specialized that later evolved enzymes (Chisholm et al., 2024), including those involved in antibiotic production and resistance. We cannot exclude the possibility that a process of exaptation (Gould and Vrba, 1982) occurred, such that the original functions of the primordial enzymes were repurposed for their current, more specialized functions.

In summary, two distinct periods are characterized by anoxic and progressively more oxygenated landscapes. The first created conditions for multimolecular aggregation and the evolution of proto-antibiotics and proto-antibiotic-resistance molecules (molecular wars). The second, based on the previously selected molecules, facilitated the formation of polyketides and more mature, complex molecules, increasing their stability and robustness. It can be hypothesized that these molecules will constitute components of proto-organelles, involved in competition among independent organelles, later among different proto-cells (Baquero et al., 2024), and finally implicated in cellular wars, thereby evolving into antimicrobials and resistance functions. Both periods overlapped extensively, accelerating molecular differentiation and the evolution of antibiotics and antibiotic-resistance counteractive actions. In the following sections, these processes, mainly the lesser-known “molecular wars,” are developed to constitute a heuristic framework for understanding the origin of these molecules.

## Molecular war and peace

In a strictly prebiotic world, antibiotic-like molecules cannot be defined by modern criteria, that is, as agents that act by altering the structure or metabolism of cellular entities. Consequently, antibiotic resistance molecules conferred no protective function on any -non existing- organisms. Similarly, the original function, mainly self-protection against spontaneous entropic instability, of multimolecular proto-organs and proto-organelles that may have preceded organisms in the origin of life (Baquero et al., 2024) are not necessarily related with the function in an integrated organism Stability and persistence in time of mono- and multi-molecular chemical structures, that is, the features that allow Darwinian selection as a mechanism of evolution, are contingent upon specific primary and secondary folding conformations. Those conformations offering selective advantages in increased stability of molecular associations, thereby resulting in a lesser degree of degradation from physical and chemical challenges encountered in the variable ancient environment, will be selected. Selective forces, a condition for selection, challenged these ancestral molecules to persist in the face of environmental inputs (Aguanell et al., 2026). The assembly of more stable structures necessitated the recruitment of specific stabilizing molecules, which may be conceptualized as molecular nutrients. This process facilitated the emergence of more stable conformations through mechanisms analogous to non-enzymatic, spontaneous posttranslational modifications. Finally, during the RNA world period, a sequential, information-rich, replicative scaffold was being organized as structured assemblies of molecules. Such structures may give rise to predecessors of ordered metabolic pathways. Hairpin or stem-loop-stable RNA structures likely coevolved alongside highly stable amino acid sequences arranged in alpha-helix or *β*-folding conformations, providing a kind of stabilizing cooperation (Wang and Du, 2025). In this stabilizing process, the co-evolution of such molecules with primordial membranes, composed of lipid bilayers and membrane proteins and already possessing membrane bioenergetics, was critical (Deamer et al., 2002; Mulkidjanian et al., 2009). Given the already-mentioned large number and diversity of molecules available in the primordial world, it can be expected that a multitude of nearly equivalent successful, stable molecular arrangements may have been environmentally selected in different, occasionally neighboring places that offer the same array of simple components. In that case, the fitness differences among the emerging molecular complexes, frequently phylogenetically related, and/or among proto-organs are likely insufficient to ensure the dominance of a few by independent selection based solely on stability or more efficient production reactions. Instead, the most effective selective events may depend on direct competition: debilitation or destruction of competitors, or interference with their “synthetic” or associative dynamics. That is, from molecular wars. However, war is compensated for by multiple molecular cooperations and individual defense. That is the essence of life.

## The molecular wars: weapons and defenses

As stated above, Julian Davies proposed that antibiotics may be among the oldest biomolecules. However, conventional wisdom holds that the biosynthesis of antibiotics requires enzymatic functions (i.e., see below for *β*-lactams), but this does not preclude the possibility that non-enzymatic molecules can interfere with the formation of prebiotic stable molecules, molecular building block assemblies, and proto-organs. For instance, amino acid condensation processes occur spontaneously under prebiotic conditions; an example concerns depsipeptides, primordial peptide backbones containing both peptide and ester bonds, probably selected by their stability (Fisher et al., 2025). These primitive, self-assembled peptides, predominantly composed of proteinaceous amino acids (Frenkel-Pinter et al., 2019), may bind to specific molecular building blocks, which may act as anti-proto-cell antibiotics. Proto-ribosomes may have contributed to the formation of depsipeptides, as ribosomes catalyzed ester bond formation (Fahnestock et al., 1970). However, prebiotic assembly of monomers may produce lactones and lactams (Chandru et al., 2020). In all cases, we should insist on the difficulty of distinguishing antibiotic actions from regulatory functions (Linares et al., 2006). In either case, these actions paved the way for the evolutionary processes.

Although we cannot have direct evidence that identifiable ancestors of modern antibiotic or resistance systems already operated in this era, the later tight association between secondary-metabolite biosynthesis and self-protection mechanisms suggests that early enzymatic networks were already navigating, exploring a chemical space in which “action” and “reaction” were intrinsically intertwined at the level of molecular assemblies. For millions of years, prebiotic random chemistry and selection for molecular stability likely coexisted with the formation of the first accelerating biochemical processes driven by the emergence of enzymatic functions. These first enzymes were simple proteins endowed with particular motifs such as phosphate-binding loops (P-loops), the seeds of NTPase enzymes, which hydrolyze nucleoside triphosphates, such as ATP. This critical step in the history of life, perhaps preceded by catalysis mediated by solid-state transition metals, which provided high-energy for the construction of novel molecules by metabolic or polymerization reactions (Romero Romero et al., 2018; Coggins and Powner, 2017). We hypothesize that among the myriad of novel molecules resulting from the action of the earliest evolving enzymes were those related to the early biosynthesis of antibiotics, antibiotic-resistance molecules, and proto-organelles of the bacterial cell, all involving prebiotic molecules and biopolymers.

## Molecular weapons and defenses in prebiotic world

As previously stated, the possibility of the emergence of proto-antibiotic and proto-resistance molecules in the prebiotic world, as elements involved in “molecular wars,” is considered in the present work. Due to brevity constraints, we restrict our examples to five major classes of antimicrobial agents. In each case, we briefly recapitulate the molecular structure of the antibiotic, the possibility of its genesis in abiotic times, the mode of action and presumed prebiotic targets, as well as the possibility of the emergence of concurrent primordial resistance molecules that protect their prebiotic targets.

### *β*-lactams, penicillin-binding-proteins, and *β*-lactamases

The ancient, highly reactive *β*-lactam ring is a four-membered cyclic structure, a 2-azetidinone, the key natural precursor of penicillins, cephalosporins, clavams, carbapenems, and monobactams; 2-azetidinones have different inhibition activities on several enzymes (Mehta et al., 2010). The antibacterial activity is based on the fact that the ring mimics the D-ala-D-ala peptidoglycan dipeptide, so that the penicillin-binding-proteins (PBPs) in charge of cell wall construction are inhibited by the analogous wrong target. The *β*-lactam ring is the result of a non-spontaneous condensation of three amino acids (*δ*-(l-*α*-aminoadipyl)-l-cysteinyl-d-valine), and is non-ribosomally synthesized by the ACV multimodular synthetase (ACVS), a complex of ribosomally-produced enzymes (Byford et al., 1997; Tahlan et al., 2017). Prebiotic *β*-lactam ring may have emerged in the “Thioester World,” roughly 4 billion years ago, as we discuss in the next paragraph. The final formation of the *β*-lactam ring involves an oxidoreduction step mediated by isopenicillin N synthase (IPNS). Some peptide precursors to antibiotics are biosynthesized this way, and later post-translational modifications produce the active antibiotic structure.

The *β*-lactam ring contains a cyclic amide bond (C1-N), the substrate of *β*-lactamases. Counterintuitively, no other biochemical structures containing *β*-lactam rings have been detected -have they existed?- so far in nature, except for *β*-lactam antibiotics (penicillins, cephalosporins, clavams, carbapenems, and monobactams), related compounds (nocardicin, tabtoxin), and probably other 2-azetidinone derivatives with enzyme-inhibiting properties. The possibility of a prebiotic *β*-lactamase function remains obscure. In the “Thioester World,” esterases may certainly have played a role, and such a function has been retained in some *β*-lactamases (Cea-Rama et al., 2022), suggesting that earlier *β*-lactams would have been formed through spontaneous ester bonds. Also, phylogenetic molecular reconstructions might cast some light on this question. In fact, the widespread serine-*β*-lactamases are phylogenetically related to the cell wall DD-transpeptidases, the high molecular weight PBPs (Fröhlich et al., 2021). With only three substitutions in the active site, PBP2X acquires *β*-lactamase activity (Peimbert and Segovia, 2003). On the contrary, low-molecular-mass PBPs exhibit some *β*-lactamase activity Both high molecular weight and low molecular weight proteins probably arose in multiple, independent evolutionary events and compete to be stabilized and become pervasive during evolution by their association with ancestral muropeptide chains, thereby diverging functionally into PBPs and *β*-lactamases (Henderson et al., 1997; González-Leiza et al., 2011). When the enzymatic action of ACVS was established, and *β*-lactam antibiotics with significant activity emerged, this divergence widened but led to a cooperative outcome. *Β*-lactamases preserved the function of PBPs in the presence of *β*-lactams, thereby allowing cell wall construction.

### Glycopeptides

D-ala–D-ala residues from lipid II are incorporated into the cell wall to form the disaccharide-pentapeptide, assuring the cross-linking of the muropeptide structure. This dipeptide is the target of glycopeptide antibiotics (e.g., vancomycin or teicoplanin), which inhibit polymerization by steric hindrance. As with ACVS in *β*-lactams, glycopeptides are non-ribosomally synthesized by biosynthetic gene clusters (BGCs) that contain non-ribosomal peptide synthetases (NRPSs). The primordial BGCs evolved to encode enzymes producing a glycopeptide precursor, paleomycin (Hansen et al., 2023). The essential peptide scaffold of glycopeptides, forming the core aglycone structure, is composed of proteinogenic (Asn and Leu) and non-proteinogenic amino acids (dihydroxyphenylglycine, hydroxyphenylglycine, and *β*-hydroxytyrosine), possibly present in the prebiotic Earth environment (Frenkel-Pinter et al., 2020a,b). We cannot exclude the possibility of prebiotic precursors of the mature glycopeptide, as the core peptidic aglycone can bind D-ala-D-ala, and thus could have some antibiotic activity. BCGs will, in any case, optimize their formation. The resulting peptide undergoes intramolecular cyclizations and enzymatic modifications, both mediated by BGCs, which are also involved in the synthesis of specialized amino sugars from glucose, arabinose, and mannose. The modified peptide, now associated with the aminosugars, can form five hydrogen bonds with the terminal D-ala-D-ala residues of the peptidoglycan precursor, inhibiting polymerization by steric hindrance and preventing muropeptide cross-linking (Waglechner et al., 2019). Resistance to glycopeptides is considered contemporary to the cellular antibiotic synthesis, both dating to 100–400 My. (Waglechner et al., 2019). In fact, most glycopeptide BGCs include the glycopeptide-resistance *vanHAX* genes to ensure self-resistance of the producer strains. As in the case of *β*-lactams, downregulators of antibiotic synthesis by BCGs could have preceded or been contemporary with the evolution of specific mechanisms of resistance, such as Van determinants (Waglechner et al., 2019). However, it cannot be ruled out that some antibiotic activity precedes the sugar decoration of the aglycone (that allows the binding to D-ala-D-ala) (Malabarba and Ciabatti, 2001). This could promote the evolution of resistance genes through multiple gene-acquisition events. Antibiotic resistance is mainly mediated by reprogramming of the pathway producing the pentapeptide target by Van determinants, disrupting the hydrogen bonds involved in drug binding and promoting electrostatic repulsion, using ligases to synthesize D-Ala-D-serine, or, providing higher resistance levels, D-ala-D-lactate, thus replacing the usual D-Ala-D-Ala target dipeptide (Arthur and Courvalin, 1993; Stogios and Savchenko, 2020). The Van gene clusters with the core genes involved in alternative systems, such as *vanA/B/D/F/M* ligases*, vanH* dehydrogenase, and *vanX* dipeptidase, hypothetically originate from lineages of glycopeptide-producing actinomycetes, but the regulatory genes (*vanRS*) are thought to emerge in *Bacillus* and *Paenibacillus* (Kardos et al., 2024; Yushchuk et al., 2020). As suggested above, the aglycone may exert an antibiotic effect through D-ala-D-ala binding, as in natural abiotic depsipeptides (D-ala-D-lac) (Frenkel-Pinter et al., 2019). Such may have been involved in some primordial resistance effect.

### Macrolides

Macrolide antibiotics are secondary metabolites derived from polyketide compounds with a macrocyclic lactone ring. There is no information about related compounds or components of the macromolecule in pre-cellular, abiotic conditions. Most of what we know about macrolide biosynthesis relates to their production in Actinomycota. The synthesis of polyketides involves a group of enzyme activities called polyketide synthases (PKSs) small carboxylic acids such as acetate and propionate into structurally diverse polyketides (Risdian et al., 2019). In *Streptomyces*, the megaenzyme Type I PKS, integrating three modules and 15 domains, and involving components such as acyl carrier protein, acyltransferase, ketosynthase, ketoreductase, dehydratase, and enoylreductase, is responsible for the production of the macrolactone (Risdian et al., 2019). PKS constructs the macrolactone (as 6-deoxyerythronolide B) through the condensation and reduction of the precursors propionyl CoA and methylmalonyl CoA, and the final macrolactone structure is controlled by alternative modular PKS (Xue and Sherman, 2000). Evolutionary intermediates have been described between PKS components (Feng et al., 2022). Whether parts of this complex construction of macrolactones could have emerged in the prebiotic world is unknown, but PKSs may have accelerated this early synthesis. Most importantly, the polyketide is decorated with a group of deoxysugars, mostly aminosugars. In the classic view, macrolides inhibit protein synthesis by targeting the nascent peptide (typically 3–10 amino acids in length) as it traverses the exit tunnel of the large ribosomal subunit. However, protein synthesis is not universally abolished; rather, inhibition occurs particularly for a subset of proteins, the so-called macrolide-sensitive proteins. It can be hypothesized that the synthesis stops because macrolides prevent the ribosome from catalyzing peptide bond formation in proteins that contain macrolide-arrest-motifs (MAMs), which are frequently Arg/Lys-X-Arg/Lys sequences. In that case, macrolides form macrolide-stalled ribosomal complexes, followed by consistent nucleotide rearrangement near the peptidyl-transferase active site (Vázquez-Laslop and Mankin, 2018). It is unclear if macrolides or their biosynthetic ancestors can interact with protoribosomes (Bose et al., 2022). The peptidyl-transferase center is essentially a ribozyme that originated in the prebiotic RNA-world hypothesis, based on the presumption that RNA could have gained the ability to facilitate the synthesis of, initially, small peptides (Müller et al., 2022). The macrolide precursor molecules that could bind to RNA in pre-cellular times are most likely C5-linked 4,6-dideoxyaminosugars, such as desoxamine, which could attach via the 2′-hydroxyl group to the N1 atom of the nucleotide A2958. Possibly later, hydrophobic interactions of the macrocyclic lactone ring could have contributed to consolidate binding to 23S rRNA, involving the base-paired nucleotides 2,611 and 2057. In summary, it cannot be discarded that in prebiotic times the function of protoribosomes could have been affected by these macrolide ancestor components, and thus the emergence of mechanisms of molecular resistance could have started to evolve.

A major mechanism of macrolide resistance is the SAM-dependent methylation or dimethylation (using S-adenosylmethionine) of the target nucleotide A2058 in the 23S rRNA. which interacts with the C5 position of the macrolactone ring. The N1 nitrogen atom of adenosine 2058 is connected by a direct hydrogen bond to the hydroxyl 2’OH at the C5 position. The expression of the methylase, encoded by the resistance gene *ermB* (among other *erm* genes) is regulated by a post-transcriptional attenuation mechanism. This occurs as a result of macrolide-ribosome stalling, which influences the translation of the leader peptides ErmBL, ErmBL2, or ErmCL preceding the co-transcribed methylase sequence in the *erm-*containing mRNA (Wang et al., 2021). Ribosome stalling destabilizes the inhibitory stem-loop mRNA structure, which is folded into secondary structures (hairpins) and exposes the Shine-Dalgarno (SD) sequence, thereby inducing the methylase (Wang et al., 2021). A similar attenuation mechanism confers weak macrolide resistance mediated by HflXR, a ribosome-splitting GTPase (Duval et al., 2018). How this ubiquitous mechanism in ribosomal functioning has evolved remains unknown. Ribosome transcriptional attenuation is also sensitive to amino acid limitation, and we can hypothesize a primordial link between those functions (Elf and Ehrenberg, 2005).

### Tetracyclines

Tetracyclines and fatty acid biosynthesis are highly related. Tetracycline’s polyketide core is modularly assembled from acetate-derived malonyl-CoA (carboxylated acetyl-CoA) and eight two-carbon fragments. This structure is thus based on an acetate-derived sequence rather than an amino acid sequence, synthesized by enzymes not distantly related to the fatty acid synthase (FAS). It iteratively inserts two-carbon units, resulting in a molecule more akin to a lipid than a peptide. The polyketide chain cycles, ultimately forming four linearly fused six-membered hydrocarbon rings. This backbone is derived from a polycyclic naphthacene-carboxamide nucleus, sometimes described as a hydrogenated naphthacene or hydronaphthacene skeleton, which is then submitted to post-assembly modifications. In any case, there are analogies between biotic and prebiotic synthesis of fatty acids, but the prebiotic synthesis relies on the use of formic and oxalic acids (Clardy et al., 2009). Advances in prebiotic lipidomics, including integrated lipidomics and transcriptomics, which have been proposed as lipotranscriptomics (Fiore et al., 2022), might shed light on potential prebiotic tetracycline ancestors. These prebiotic tetracycline ancestral components, particularly their lipophilic components, could have contributed to the stabilization of RNA conformations, thereby altering ribozyme activity (Czerniak and Saenz, 2022). It is well-established that tetracyclines inhibit translation by binding to nucleotides in helix 34 and helix 31 of the 16S rRNA, thereby preventing the binding of aminoacyl-tRNA to the mRNA-ribosome complex, which probably is a prebiotic ribozyme (Chukwudi, 2016; Suga et al., 2011). Therefore, we may hypothesize that tetracycline ancestors contributed to the dynamics of competitive interaction among proto-ribosomes.

In this prebiotic scenario, the main mechanism of tetracycline resistance, efflux pumps, as we understand by now the function of these structures (necessarily associated with cell membranes), was probably irrelevant. It has recently been suggested that translation factors likely emerged early, providing a bridge between pre-cellular chemistry and the last universal common ancestor (LUCA) (Fer et al., 2025). Primordial translation factors are likely ancestors of ribosomal protection proteins (RPPs), producing conformational changes in the ribosomal RNA and, mediating tetracycline resistance. Indeed, these RPPs share structural similarity with translation factors EF-G/EF-2 and EF-Tu/EF-1α (Kobayashi et al., 2007).

### Aminoglycosides

Aminoglycosides are composed of two or three amino sugars, linked by glycosidic bonds to a core amino-substituted cyclohexane amino cyclitol (a cyclic alcohol), as streptidine in the case of streptomycin, or 2-deoxystreptamine, for other aminoglycosides. The amino cyclitols are substituted by aminosugars, such as neomycin (4,5-disubstituted) or kanamycin, tobramycin, and gentamicin (4,6-disubstituted). We can hypothesize that prebiotic synthesis of the aminoglycoside precursor monosaccharides originates from the formose network in basic aqueous solution and in nonaqueous conditions (Lamour et al., 2019; Yi et al., 2023). Aminocyclitol molecules can be formed in prebiotic conditions from glycolaldehyde, 2-aminooxazole, and aminonitrile (Whitaker and Powner, 2022). Therefore, the formation of precursors of highly positively-charged aminocyclitols-aminoglycosides in the pre-cellular world cannot be ruled out, as well as their possible interactions with negatively-charged RNA in proto-ribosomes, particularly their ribozyme cores. In the cellular world, Actinomycetes optimized the biosynthetic production of aminocyclitols from simple sugar units (as D-glucose-6-phosphate), using sugar phosphate cyclases (SPCs) (Kudo and Eguchi, 2022; Kudo and Eguchi, 2009) including the 1 L-*myo*-inositol 1-phosphate (MIP) synthases and the 2-deoxy-*scyllo*-inosose synthases involved in the biosynthesis of pseudo trisaccharide intermediates, and finally aminoglycoside antibiotics (Mahmud, 2009; Chen et al., 2025; Wehmeier and Piepersberg, 2009). The biosynthesis of the different aminoglycosides follows parallel pathways, likely reflecting a rooted evolutionary tree (Yu et al., 2017; Kirby, 1990). That might explain why aminoglycoside-producing strains frequently biosynthesize several aminoglycoside molecular variants. In contrast to the multistep enzymology presented above for the biosynthesis of polyketides (or non–ribosomal peptides) in other antibiotic groups, here the biosynthetic pathways are largely based on monofunctional enzymes catalyzing single steps.

Aminoglycosides bind the decoding region at the A site of the 30S ribosomal subunit, disrupting bacterial translation. These antibiotics are multiple positively charged compounds. The positive charges are attracted to the negatively charged phosphate RNA backbone. The flexibility of aminoglycosides facilitates accommodation in binding pockets within internal loops of RNA helices or within ribozyme cores to form specific contacts (Schroeder et al., 2000). Binding involves the amino and hydroxyl functional groups of aminoglycosides and RNA bases. The presence of 2-deoxystreptamine (2-DOS) in almost all aminoglycosides indicates a key role in RNA recognition and biological activity (Chittapragada et al., 2009). The structural modification of the antibiotic by the aminoglycoside-modifying enzymes *N*-acetyltransferases (AACs), *O*-phosphotransferases (APHs), and *O*-nucleotidyltransferases (ANTs), as well as the blocking-binding methylases of the antibiotic decoding center on the 16S rRNA (Arm/Rmt and Kam/Npm methylases), comprise the major mechanisms of aminoglycoside resistance present in the antibiotic-producing organisms. The origin of aminoglycoside resistance genes in the cellular biosynthetic machinery of these compounds has been known for a long time (Distler et al., 1985). The question is whether prebiotic aminoglycoside-resistance ancestors existed before the emergence of cellular life. The sugar acetyltransferase function may have emerged in the prebiotic world, using high-energy compounds such as acetyl phosphate or thioesters as acetyl-CoA precursors in the hypothetic “Thioester World,” considered a key period in the origin of life (Singh et al., 2025). Prebiotic phosphotransferases are also possible: for instance, sugar-1-phosphates can be produced spontaneously in mixtures of sugars and phosphoric acid (Nam et al., 2017). Prebiotic sugar nucleotidyltransferases facilitate the transfer of a nucleotide from a nucleotide triphosphate to a sugar, and may have originated in promiscuous ribozymes, in the mixed transitional period of the Thioester World and the later RNA world, and could be related to the formation of sugar nucleotides (Biscans, 2018). rRNA methylation was also available in the prebiotic world (Schneider et al., 2018). If the molecules associated with these RNA-modifying structures have any effect on the interactions of aminocyclitol or aminoglycoside ancestors and could constitute the basis for the later evolution of antibiotic resistance enzymes, it remains to be explored. However, the early prebiotic Earth was endowed with the basic constituents of these protective functions.

## Junction and disjunction between antibiotic production and resistance

According to the hypothesis of molecular wars discussed in the previous paragraphs, an extensive prebiotic period likely existed during which molecules capable of destabilizing rival molecular assemblies emerged. Concurrently, compensatory molecules appeared, functioning to shield these structures from such proto-antibiotics. The evolution of molecular ensembles and pathways responsible for generating these inhibitory agents may have been intrinsically linked to the partial self-destruction of their own synthetic pathway. Consequently, the evolutionary association with protective molecules would have conferred a significant selective advantage, as unprotected target molecules are supposed to be systematically eliminated when present in the same environment. In subsequent evolutionary stages, the synthesis of proto-antibiotics may have been integrated into a comprehensive self-protection strategy. Such an association persisted in later cellular secondary metabolism biosynthetic processes. As previously stated, in Actinomycetota (also in Bacillota), resistance molecules that inactivate the produced antibiotic are frequently present, and the chemical structure of these detoxifying mechanisms usually derives from the same biosynthetic pathways of antibiotic secondary metabolism. However, some strains harbor antibiotic-resistance mechanisms against antibiotics they do not produce. This is similar to many other non-antibiotic-producing bacteria that harbor resistance mechanisms to antibiotics used in antimicrobial therapy. Note that, beyond specific mechanisms that inactivate the antibiotic, resistance can result from a refractory structure of the antibiotic target or from negative regulation of antibiotic biosynthesis when the bacteria are damaged. This strongly suggests that resistance genes have been disjoined from the biosynthetic ones, probably captured by gmobile genetic elements such as integrons, transposons, or integrative-conjugative elements, and eventually harbored in transmissible plasmids. This may give rise to horizontal gene transfer of these resistance genes, which may eventually be acquired by organisms, taxonomically related or unrelated to antibiotic producers, as long ago predicted by Julian Davies and Arnold Demain. However, natural constraints on the mobility of mobile genetic elements impede a pervasive introduction of antibiotic resistance genes into the microbial world (Waglechner and Wright, 2017).

Our modern anthropocentric intuition and education tend to equate antibiotics with drugs and, consequently, to assume that they act primarily as lethal weapons in “microbial wars.” However, for any released bioactive molecules, concentration and context are critical: in natural environments, the amount of antibiotic produced by individual cells or small populations is rarely sufficient to ensure the killing of potential competitors, but can profoundly modulate transcriptional programs and community dynamics at sub-inhibitory levels. In this ecological setting, antibiotics are better understood as signaling or regulatory molecules, whose coordinated production and corresponding resistance mechanisms shape interaction networks and niche occupancy, rather than as simple bactericidal agents (Linares et al., 2006; Davies, 2006; Aminov, 2009; Sengupta et al., 2013). Large-scale anthropogenic production and dissemination of antibiotics have disrupted this long-standing balance between production and resistance, creating pervasive, strong selection for resistance determinants that can spread widely by horizontal gene transfer. In evolutionary terms, this massive and sustained selection does not “aim” to preserve an ancient equilibrium but, blindly, favors mobile genetic elements and bacterial lineages in which resistance functions maintain cellular viability under antibiotic exposure. We suggest that resistance, as a reaction mechanism, contributes to maintaining some form of equilibrium, ensuring healthy diversity in the microbiosphere and preventing the extinction of ecological nodal lineages. In our view, this process was rooted in prebiotic molecular war and peace dynamics.

## Conclusion

**Figure 1:**
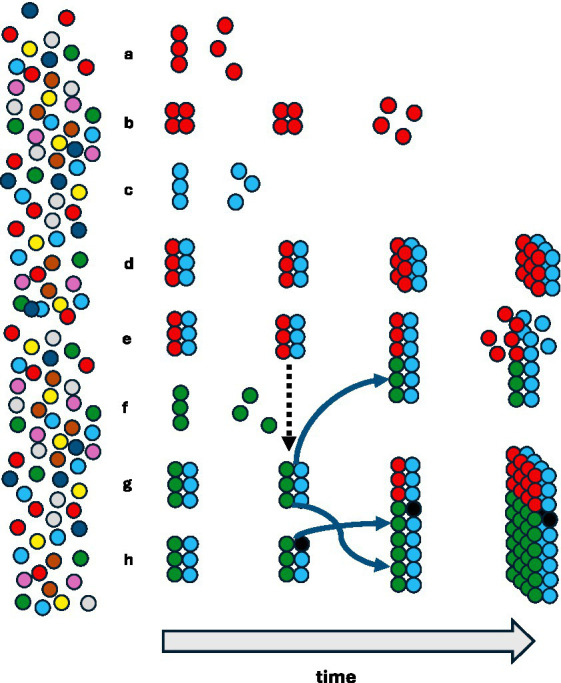
At the left, the disordered probiotic molecular soup. Eventually, some of these molecules may associate. **(a)** a triplet (red) is formed, but it is unstable; **(b)** a 4-membered structure is formed, which is more stable in time, but after a period the stability is lost; **(c)** another triplet is formed, slightly more stable than **(a)**, but also transiently; **(d)** the triplet in **(a)** associates with the blue triplet in **(c)**, and such structure becomes stable in time, and even is able to replicate; **(e)** such successful process is interfered by a further association with another molecular association, an antimolecule –or proto-antibiotic- which disassociate the **(d)** structure; note that (dashed arrow) this association might facilitate the antimolecule formation; **(f)** again, a (green) triplet is formed, but it is unstable; **(g)** stability is gained by association with the blue triplet, so that both triplets in association becomes a stable structure; this is the antimolecule acting in **(e)**; **(h)** a variation of the antimolecule, now with a black member that does not influence stability, integrates in the successful structure **(d)**, and now the antimolecule cannot disturb the successful replication of **(d)** as antimolecule is not active any more (dashed white lines).

Our understanding of the origins of antibiotics and antibiotic-resistance genes remains nascent. However, we propose that both cases may be traced back to prebiotic times, arising from a primordial field of action and reaction dynamics. In this environment, ancestral molecules competed through processes of disaggregation and cooperation to sustain their structural persistence over time (Figure 1). Such molecular competition (“molecular wars”) could have exerted a powerful effect on the evolution of life during a prolonged, fuzzy transition period, when pre-cellular and post-cellular worlds coexisted. The mechanisms governing these molecular wars could have been instrumental in the foundational stages of life, for instance, providing weapons and defenses in the hypothetical proto-organs/organelles wars before the emergence of organisms (Baquero et al., 2024). Within the framework of the “virus-first” theory, which posits the existence of proto-viruses in a ribonucleoprotein world (Prosdocimi and de Farias, 2025), some of these early antibiotics and defenses could have played a role in the hypothetical wars opposing pre-cellular and post-cellular entities. In the end, a kind of armistice, characterized by cooperation without completely losing the confrontation, could have evolved, and this situation does not contradict the structure of the current natural microbial world. This arrangement was also possible because the antibiotics and antibiotic-resistance genes were mainly confined to bacterial lineages whose lifestyles (mostly sporogenic organisms) required the production of antibiotics in a strictly necessary way. Only the anthropogenic intervention of industrial production of antimicrobial agents, providing a massive selective force for resistance, has altered this armistice, facilitating the spread of antibiotic resistance genes via mobile genetic elements.

The hypothetical framework presented in this work concerning the prebiotic origin of anti-structural and structure-protective agents, which may, respectively, evolve into antibiotics and antibiotic resistance mechanisms, could provide a fertile field of research. First, to understand the origins of functions in the microbial world; second, to seek undiscovered, potentially useful functions, including novel polyketides that could yield new antimicrobials. That is particularly relevant in our time, under the global threat of antimicrobial resistance.

We should be mindful of the complexity of developing experimental models based on the geochemical conditions of prebiotic Earth. This will require a massive international investment to identify at least some pieces of what we have lost over billions of years of molecular evolution. A future mega-Miller-Urey experiment (1953) could be based on geochemical simulations using our current advanced knowledge of the building blocks of life, as well as the automated exploration of prebiotic chemical spaces (Kitadai and Maruyama, 2018; Sharma et al., 2021). A number of useful principles can be derived from the exploration of natural biosynthetic biology.
